# Potential Antifungal Targets Based on Glucose Metabolism Pathways of *Candida albicans*

**DOI:** 10.3389/fmicb.2020.00296

**Published:** 2020-03-17

**Authors:** Xueqi Chen, Zewen Zhang, Zuozhong Chen, Yiman Li, Shan Su, Shujuan Sun

**Affiliations:** ^1^Department of Pharmacy, Shandong Provincial Qianfoshan Hospital, Shandong University, Jinan, China; ^2^School of Pharmaceutical Sciences, Shandong University, Jinan, China; ^3^Department of Imaging Medicine and Nuclear Medicine, Qilu Medical College, Shandong University, Jinan, China; ^4^Department of Pharmacy, Zibo Central Hospital, Zibo, China; ^5^Department of Clinical Pharmacy, The First Affiliated Hospital of Shandong First Medical University, Jinan, China

**Keywords:** *Candida albicans*, glucose metabolism pathways, potential antifungal targets, growth, virulence

## Abstract

In recent years, fungal infections have become a serious health problem. *Candida albicans* are considered as the fourth most common isolates associated with approximately 40% mortality in bloodstream infections among hospitalized patients. Due to various limitations of classical antifungals used currently, such as limited kinds of drugs, inevitable toxicities, and high price, there is an urgent need to explore new antifungal agents based on novel targets. Generally, nutrient metabolism is involved with fungal virulence, and glucose is one of the important nutrients in *C. albicans*. *C. albicans* can obtain and metabolize glucose through a variety of pathways; in theory, many enzymes in these pathways can be potential targets for developing new antifungal agents, and several studies have confirmed that compounds which interfere with alpha-glucosidase, acid trehalase, trehalose-6-phosphate synthase, class II fructose bisphosphate aldolases, and glucosamine-6-phosphate synthase in these pathways do have antifungal activities. In this review, the glucose metabolism pathways in *C. albicans*, the potential antifungal targets based on these pathways, and some compounds which have antifungal activities by inhibiting several enzymes in these pathways are summarized. We believe that our review will be helpful to the exploration of new antifungal drugs with novel antifungal targets.

## Introduction

In recent years, invasive fungal infections have been increasing all the time due to the widespread use of immunosuppressants and antibiotics, the increase of patients with AIDS, and the development of organ transplantation (Van Ende et al., 2019). The rate of *Candida albicans* isolation ranks fourth and the mortality approximately ups to 40% among patients with bloodstream infections (Yesilkaya et al., 2017). *C. albicans* infections have become a challenge in clinic (Yesilkaya et al., 2017). The clinically used antifungal agents are confined to several major types, such as azoles, which inhibit ergosterol biosynthesis, polyenes that combine with ergosterol in the fungal cell membrane, echinocandins that inhibit β-1,3-glucose biosynthesis, and flucytosine that inhibits DNA synthesis (Zavrel and White, 2015). As there are many limitations in clinical application of these antifungal agents, such as limited available drugs, inevitable toxicities, high price, and the emergence of drug resistance, the development of safe and effective antifungal drugs based on novel antifungal targets is urgently needed (Cuenca-Estrella, 2014; Zavrel and White, 2015).

In case of the further researches of fungal pathogenicity, many new antifungal compounds with novel antifungal mechanisms have been identified. Cationic antimicrobial peptides are proved to kill fungi by changing the structure of cell membrane lipid bilayer (Yeaman and Yount, 2003). E1210 can inhibit fungi catalytic enzymes of GPI-anchored protein synthesis pathways (Hata et al., 2011). The Hsp90 inhibitors, such as Mycograb, show antifungal effects by inhibiting Hsp90 (Matthews et al., 2003; Cordeiro Rde et al., 2014).

Besides, previous studies have shown that nutrient metabolism disorders are closely related to fungal virulence (Jiang and Pan, 2018; Rutherford et al., 2019). All microorganisms rely on obtaining sufficient nutrients to produce energy to maintain cellular homeostasis, and glucose is one of the important nutrients, thus researches on glucose metabolism have also been focused (Lorenz, 2013).

**FIGURE 1:**
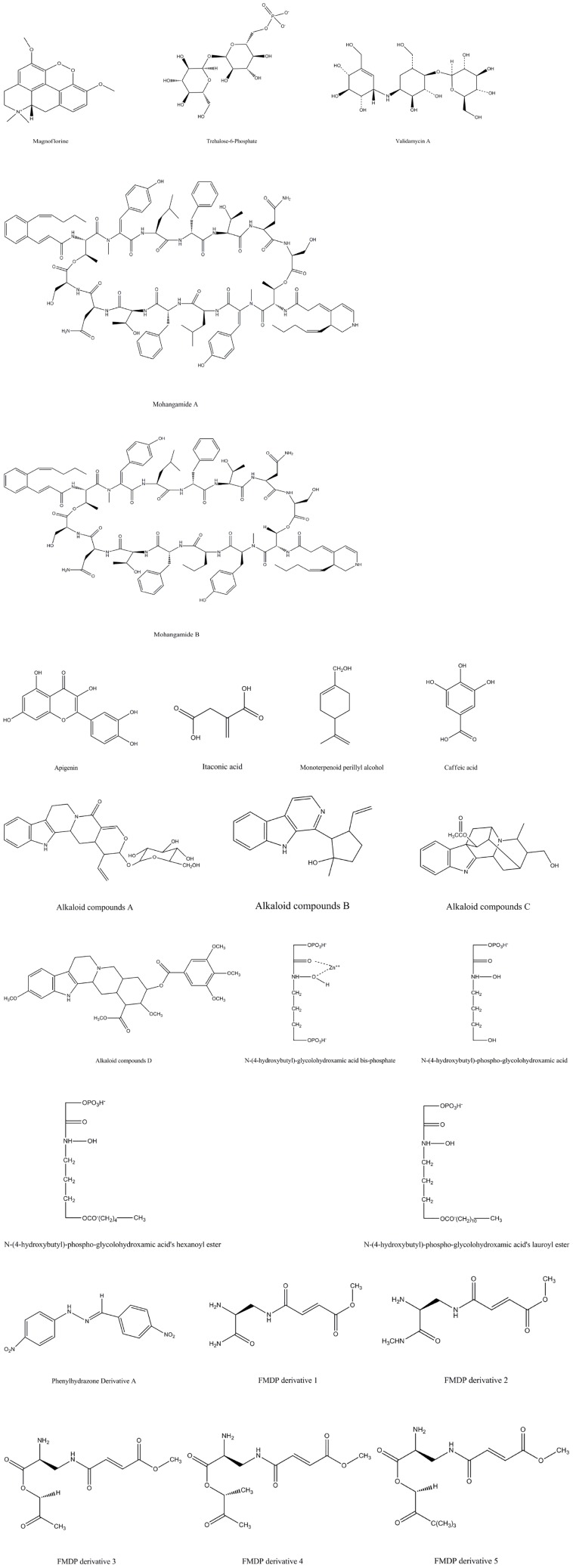
The structures of compounds against *Candida albicans* by interrupting with glucose metabolism pathways.

*C. albicans* can obtain glucose from a variety of pathways, such as being transported into cells by glucose transporters, and being synthesized by polysaccharide hydrolysis pathway, gluconeogenesis, trehalose biosynthesis pathway, and galactose metabolism (Bramono et al., 1995; Zaragoza et al., 1998; Eschrich et al., 2002; Barelle et al., 2006; Singh et al., 2007; Lorenz, 2013). *C. albicans* also can metabolize glucose to gain energy through glycolytic pathway and TCA cycle (Barelle et al., 2006; Lorenz, 2013). All of these glucose metabolism pathways in *C. albicans* involve a lot of intermediates that are catalyzed by corresponding enzymes (Barelle et al., 2006). Especially when *C. albicans* invades human bodies, it must adapt to distinct glucose concentrations in different human niches (Van Ende et al., 2019). When *C. albicans* encounters the glucose-shortage environment, such as macrophages or neutrophils, the genes of fatty acid β-oxidation, glyoxylate cycle, and gluconeogenesis are induced, *C. albicans* is able to synthesize glucose through these pathways (Barelle et al., 2006; Brown et al., 2006, 2007; Rodaki et al., 2006; Ramirez and Lorenz, 2007; Strijbis and Distel, 2010). Whereas *C. albicans* exists in plasma and tissues after escaping from the macrophages or neutrophils, the genes of glycolytic pathway and TCA cycle are induced, and *C. albicans* could resume these pathways to gain energy (Barelle et al., 2006; Brown et al., 2006, 2007; Rodaki et al., 2006; Ramirez and Lorenz, 2007; Strijbis and Distel, 2010). There are several fungal specific enzymes among these pathways such as acid trehalase, trehalose-6-phosphate synthase, trehalose-6P phosphatase, enolase, class II fructose bisphosphate aldolases, pyruvate kinase, and glucosamine-6-phosphate synthase which are closely related to *C. albicans* virulence, and researches have demonstrated that inhibitors against these specific enzymes have great application prospects against candidiasis (Barelle et al., 2006). In the following review, we are primarily focusing on the antifungal activities of some compounds with the potential antifungal targets on fungal specific enzymes in glucose metabolism pathways. *C. albicans* glucose metabolism pathways and potential antifungal targets based on these pathways are illustrated in Figure 1. The compounds which have the antifungal activities by disrupting the enzymes of these pathways are summarized in Table 1. We believe this review can pave the way for the emergence of new antifungal drugs in the future.

**TABLE 1:** Antifungal activities of compounds against *Candida albicans* by interrupting with glucose metabolism pathways.

Antifungal compounds	Strains	Antifungal activities	Potential antifungal targets	References
Magnoflorine (MF)	*C. albicans ATCC10231*	When MF used alone: MIC = 50 μg/ml; the MIC value of miconazole decreased from 3.125 to 1.5625 μg/ml when combined with 6.25 μg/ml MF	Alpha-glucosidase	Kim et al., 2018
Trehalose-6-phosphate	*C. albicans ATCC 36802*	125 μM can inhibit 50–60% TPS1 activity of *Candida albicans*	Trehalose-6-phosphate synthase in trehalose pathway	Magalhaes et al., 2017
Validamycin A	*C. albicans CEY-1*	MIC_50_ = 500 μg/ml	Trehalase in trehalose pathway	Guirao-Abad et al., 2013
Mohangamide A	*C. albicans*	IC_50_ = 4.4 μM	Isocitrate lyase in glyoxylate pathway	Bae et al., 2015
Mohangamide B		IC_50_ = 20.5 μM		
Apigenin	*C. albicans ATCC10231*	MIC = 125 μg/ml		Cheah et al., 2014
Itaconic acid		MIC = 250 μg/ml		
Monoterpenoid perillyl alcohol	*C. albicans SC5314*	MIC_80_ = 320 μg/ml		Ansari et al., 2018
Caffeic acid	*C. albicans ATCC10231*	MIC = 1000 μg/ml		Cheah et al., 2014
Alkaloid compounds A	*Paracoccidioides* spp.	IC_50_ = 17 μg/ml	Malate synthase in glyoxylate pathway	Costa et al., 2015
Alkaloid compounds B		IC_50_ = 29 μg/ml		
Alkaloid compounds C		IC_50_ = 21 μg/ml		
Alkaloid compounds D		IC_50_ = 17 μg/ml		
*N*-(4-hydroxybutyl)-glycolohydroxamic acid bis-phosphate *N*-(4-hydroxybutyl)-phospho-glycolohydroxamic acid	*C. albicans*	IC_50_ = 0.003 μg/ml	Class II fructose bisphosphate aldolases	Daher et al., 2010
		IC_50_ = 0.3 μg/ml		
*N*-(4-hydroxybutyl)-phospho-glycolohydroxamic acid’s hexanoyl ester		IC_50_ = 0.28 μg/ml		
*N*-(4-hydroxybutyl)-phospho-glycolohydroxamic acid’s lauroyl ester		IC_50_ = 0.8 μg/ml		
Phenylhydrazone derivative A		When the compound A was used alone: IC_50_ = 2.7 μg/ml; the IC value of the compound was decreased from 2.7 to 0.0625 μg/ml when combined with 8 μg/ml fluconazole		Han et al., 2017
*N*^3^-(4-methoxyfumaroyl)-(S)-2,3-diaminopropanoic acid (FMDP) derivative 1	*C. albicans ATCC 10231*	MIC = 4 μg/ml	Glucosamine-6-phosphate synthase	Pawlak et al., 2015
FMDP derivative 2		MIC = 32 μg/ml		
FMDP derivative 3		MIC = 0.25 μg/ml		Pawlak et al., 2016
FMDP derivative 4		MIC = 0.5 μg/ml		
FMDP derivative 5		MIC = 0.5 μg/ml		

**MIC, minimum inhibitory concentration; IC_50_, half maximal inhibitory concentration.**

## Potential Antifungal Targets in Glucose Metabolism Pathways

In the following, we described the various potential antifungal targets which are proved to be related to *C. albicans* virulence in these glucose metabolism pathways, and several compounds which have antifungal activities based on alpha-glucosidase, acid trehalase, trehalose-6-phosphate synthase, isocitrate lyase, malate synthase, enolase, class II fructose bisphosphate aldolases, and glucosamine-6-phosphate synthase.

### Glucose Transporters and Sensors

Previous studies demonstrated that 20 glucose transporters (HGTs) had been identified in *C. albicans*, and interfering with some HGTs had obvious effects on hyphal formation, virulence, and drug resistance of *C. albicans* (Brown et al., 2006; Van Ende et al., 2019). As shown in Figure 1, HGT1 and HGT4 are the important glucose transporter and glucose sensor, respectively, which are required for sensing glucose, galactose, maltose, and fructose in *C. albicans* (Varma et al., 2000; Brown et al., 2006). HGT1 is a high-affinity glucose transporter in *C. albicans*, and Varma et al. suggested that the expression of HGT1 was significantly increased when used with some antifungal drugs, illustrating that HGT1 is closely related to drug resistance (Varma et al., 2000). Brown et al. pointed that the hgt4Δ mutant could not grow normally on media containing glucose, mannose, or fructose at low concentrations, and the hgt4Δ mutant had a growth defect without proper aeration even at high sugar concentration, demonstrating that HGT4 is essential for *C. albicans* growth (Brown et al., 2006). Generally, the disruption of genes of glucose transporter and glucose sensor in *C. albicans* was involved with hyphal formation and virulence, and the hgt4Δ null mutant had obvious hyphal formation defects on spider medium compared with the wild-type strains (Brown et al., 2006; Van Ende et al., 2019). Moreover, the hgt4Δ mutant also displayed less virulence compared with the wild-type strains, elucidating that HGT4 does have a great influence on *C. albicans* hyphal formation and virulence (Brown et al., 2006; Van Ende et al., 2019). Furthermore, the sequence similarities between HGT1/HGT4 in *C. albicans* and GLUTs in humans are less than 30.4% (Fan et al., 2002). Therefore, in theory, HGT1 and HGT4 have the possibilities to become potential antifungal targets, but they still need a lot of exploration to determine the feasibility.

### Alpha-Glucosidase

Alpha-glucosidase is a carbohydrate-degrading enzyme which can hydrolyze disaccharides such as maltose to glucose for subsequent glycolysis and tricarboxylic acid cycle (Bramono et al., 1995). Alpha-glucosidase I and II in endoplasmic reticulum encoded by CWH41 gene and ROT2 gene, respectively, are also indispensible for *C. albicans N*-oligosaccharide processing, which is essential for host–fungus interaction (Mora-Montes et al., 2007). cwh41Δ and rot2Δ null mutants displayed delayed filamentation and shorter germ tubes, reduced contents of mannan in cell wall, and decreased virulence in the murine model infected with cwh41Δ and rot2Δ null mutants, demonstrating that alpha-glucosidases are vital for *C. albicans* growth and virulence (Mora-Montes et al., 2007). Therefore, we believe that the novel antifungal compounds based on alpha-glucosidases can be found. As shown in Figure 1, Kim et al. (2018) pointed that 150 μM magnoflorine could completely inhibit the formation of *C. albicans* biofilm and magnoflorine has obvious antifungal activity against *C. albicans* with minimum inhibitory concentration (MIC) 50 μg/ml, and the MIC of miconazole was reduced from 3.13 to 1.56 μg/ml when combined with 6.25 μg/ml magnoflorine. In addition, the combination of miconazole and magnoflorine shows a weak synergistic anti-candida effect (Kim et al., 2018). Moreover, the cytotoxicity to human cells of magnoflorine was also tested, and it exhibited no toxicity to human cells even in 600 μM of treatment (Kim et al., 2018). Thus, magnoflorine based on alpha-glucosidases has no significant toxicity to human cells, and we hope this enzyme could become a potential antifungal target for *C. albicans* infection.

### Fructose-1,6-Bisphosphatase

Fructose-1,6-bisphosphatase (FBPase) is a rate-limiting enzyme encoded by FBP1 gene in gluconeogenesis metabolism which is responsible for converting the hydrolysis of fructose 1,6-bisphosphate to fructose 6-phosphate (Kaur et al., 2017; Liu and Zhang, 2018). *C. albicans* which lack the FBP1 gene exhibited significant defects in growing on non-fermentable carbon sources, such as glycerol, acetate, ethanol, citrate, and oleate (Ramirez and Lorenz, 2007). Interfering with genes of enzymes in *C. albicans* glucose metabolism pathways is always associated with hyphae formation and virulence. Although fbp1Δ mutant had no difference in yeast–hyphae transition compared with wild-type strains, the virulence of fbp1Δ mutant strain was significantly attenuated in a mouse model with disseminated system candidiasis compared with wild-type strains, demonstrating that FBP1 gene is unnecessary for morphological transition of *C. albicans* in glucose-limited environment but is indeed essential for virulence (Eschrich et al., 2002; Ramirez and Lorenz, 2007). Furthermore, FBPase is a highly conserved enzyme in eukaryotes, but there is only 48% sequence homology between *Saccharomyces cerevisiae* and animals (Rogers et al., 1988). In summary, it is possible for FBPase to become a potential antifungal target theoretically, but more experimental researches are needed to confirm that.

### Phosphoenolpyruvate Carboxykinase

Phosphoenolpyruvate carboxykinase (PEPCKase) is generally regarded as an enzyme which converts oxaloacetate into phosphoenolpyruvate (PEP) and carbon dioxide in gluconeogenesis (Zelle et al., 2010). In *C. albicans*, pck1/pck1 null mutant grew well on glucose, but they were unable to grow on non-fermentable carbon sources (Barelle et al., 2006). Barelle et al. (2006) demonstrated that *C. albicans* pck1/pck1Δ null strain showed a moderate decrease in virulence in murine systemic infections compared with wild-type strains, suggesting that PEPCKase is indeed a contributory factor for the virulence of *C. albicans*. Moreover, *C. albicans* PEPCKase has high homology with other ATP-dependent PEPCKase, but there is no homology to the GTP-dependent PEPCKase of animals (Leuker et al., 1997). Therefore, although there are no developments on antifungal compounds based on this enzyme, these physiopathological studies have made this enzyme possible as a potential antifungal target theoretically, and more researches are needed to confirm the feasibility in the future.

### Acid Trehalase and Heat-Shock Proteins

Pedreno et al. (2004) suggested that acid trehalase (Atc1p) which is encoded by ATC1 gene is located on the cell wall surface, and it is responsible for cleaving off exogenous trehalose and growing on trehalose as a carbon source. *C. albicans* lost acid trehalase activity and could not grow on exogenous trehalose as sole carbon source when ATC1 gene was disrupted (Pedreno et al., 2004). Moreover, ATC1 gene does have influence on *C. albicans* hyphal formation and virulence. ATC1Δ mutated strain displayed a significant decrease in virulence in mice models and a considerable reduction in yeast-to-hyphae transition compared with wild-type strains (Pedreno et al., 2007). However, *C. albicans* ATC1 gene-deficient mutant was more resistant in response to various stimuli such as oxidative stress and heat shock, so the regulation of ATC1 gene in *C. albicans* is very complicated, the usefulness of Atc1p as an antifungal target remained to do more intensive researches (Pedreno et al., 2007; Sanchez-Fresneda et al., 2014). In the development of new antifungal compounds with this enzyme, Guirao-Abad et al. identified the antifungal activity of the Atc1p competitive inhibitor validamycin A, and the MIC_50_ was 500 g/l (Guirao-Abad et al., 2013). In theory, Atc1p has the possibility to be a potential antifungal target; it still needs more exploration to assure the feasibility.

In addition, heat shock proteins (Hsps) such as Hsp90 and Hsp21 are closely related to the trehalose biosynthesis of *C. albicans*. Several reports suggested that Hsp21 in *C. albicans* plays a vital role in adapting to distinct environmental stresses by modulating the homeostasis of trehalose and the activation of stress-responsive kinase Cek1 (Mayer et al., 2012). Importantly, the hsp21Δ/Δ mutant failed to grow at elevated temperature and exhibited invasive growth defects, significantly shorter filamentation, and increased susceptibility toward oxidative stress and human neutrophils compared with the wild-type strains (Mayer et al., 2012). Furthermore, hsp21Δ/Δ mutant was more sensitive to various antifungal drugs (Mayer et al., 2013; Gong et al., 2017). Many studies have demonstrated that Hsp90 established vital roles in thermal stability, hyphal formation, apoptosis, and drug resistance in *C. albicans*, and the synergistic effects against FLC-resistant *C. albicans* have also been proved when specific inhibitors of Hsp90 are combined with fluconazole (Serneels et al., 2012; Li et al., 2015; Gong et al., 2017). Therefore, the study of specific inhibitors targeting Hsp90 and the inhibitors in combination with classical antifungals could be promising strategies against *C. albicans* infections. Hsp21 has the possibility to be a potential antifungal target theoretically, but it still requires a lot of exploration to determine the feasibility.

### Trehalose-6-Phosphate Synthase

In *C. albicans*, the trehalose-6-phosphate synthase (Tps1P) encoded by TPS1 gene is required for producing trehalose, and it is responsible for catalyzing the first step in trehalose biosynthesis. Except for playing an important role in trehalose biosynthesis, Tps1p is also responsible for *C. albicans* cell viability (Alvarez-Peral et al., 2002). The tps1/tps1 null mutant of *C. albicans* lost their high cell viability during severe oxidative stress conditions induced by high concentrations of hydrogen peroxide (Alvarez-Peral et al., 2002). Importantly, the TPS1 gene was required for *C. albicans* hyphal formation and virulence, and disruption of TPS1 gene showed a noticeable hyphal conversion defect at 37°C and an obvious decrease in infectivity compared with the wild type in mice models, which means that TPS1 gene is indeed a contributory factor to *C. albicans* viability and virulence (Zaragoza et al., 1998). In different media, tps1/tps1 null mutant exhibited distinct phenotypes due to the difference in the over-glycosylation and hydrophobicity (Martinez-Esparza et al., 2007; Thammahong et al., 2017). It has been confirmed that tps1/tps1 null mutant was more vulnerable to macrophage killing when cultured in solid media, but the phenotype was reversed in liquid media (Martinez-Esparza et al., 2007; Thammahong et al., 2017). However, resistance of *C. albicans* TPS1 gene mutant to different stimulus conditions is different (Zaragoza et al., 2003). *C. albicans* TPS1 mutant was more sensitive to severe oxidative stress conditions, but in saline stress or heat shock stress conditions, the sensitivity of TPS1 mutant and the wild type had no remarkable difference (Zaragoza et al., 2003). Therefore, in *C. albicans*, the virulence and the susceptibility to stimulus conditions based on Tps1p regulation is complex; more research on the enzyme is still needed to assure if it is proper to become an antifungal target. Fortunately, Tps1p is absent in mammals, so specific Tps1p inhibitors might have little human side effects (Magalhaes et al., 2017).

In Figure 1, a new effective antifungal compound has been identified based on Tps1p; trehalose-6-phosphate (T6P) inhibited 50 and 60% activity of *Candida tropicalis* Tps1p and *C. albicans* Tps1p *in vitro* at 125 μM concentration, respectively (Magalhaes et al., 2017). Although T6P has unsuitable drug properties and lacks data on *C. albicans* virulence *in vivo*, it provides an effective design idea and the T6P analogs can be developed for novel antifungals in *C. albican*s treatment (Magalhaes et al., 2017). It still needs further developments to suggest the therapeutic potential of Tps1p inhibitors in fungal infections, and we believe Tps1p is an important enzyme for developing new anti-candida drugs.

### Trehalose-6P Phosphatase

Trehalose-6P phosphatase (Tps2p) plays an important role in trehalose biosynthesis pathway which can convert T6P to trehalose and inorganic phosphate (Thammahong et al., 2017). T6P is toxic to *C. albicans* and the disruption of Tps2p protein will accumulate T6P in cells (Thammahong et al., 2017). Importantly, Tps2p and T6P are all absent in mammals, and Tps2p is a specific enzyme to dephosphorylate T6P, so the development of specific Tps2p inhibitors is meaningful (Thammahong et al., 2017). The gene TPS2 encoding Tps2p plays a vital role in the growth and cell viability of *C. albicans*, but no obvious influence on the hyphae formation (Van Dijck et al., 2002). During the heat stress condition, the tps2/tps2 null mutant displayed the inhibition of growth and the loss of cell viability, but no difference in yeast–hyphae transition compared with the wild-type strain (Van Dijck et al., 2002). Moreover, TPS2 gene was also related to *C. albicans* virulence; the loss of TPS2 gene strongly decreased the virulence in mouse models with system infections because of the T6P accumulation in *C. albicans* (Maidan et al., 2008). The sensitivity to macrophage phagocytosis of tps2Δ/tps2Δ strain is also quite different compared with wild-type strains, and the results demonstrated that *C. albicans* strain which lacks the TPS2 gene is more susceptible to macrophage engulfment than wild-type strains (Martinez-Esparza et al., 2009). Given that Tps2p is essential for *C. albicans* growth, cell viability, and virulence, further development of specific Tps2p inhibitors can help us to identify the novel antifungals against *C. albicans* infections.

### Isocitrate Lyase

Isocitrate lyase (Icl) catalyzes isocitrate to glyoxylate and succinate in the glyoxylate cycle, then succinate is used for the subsequent glucose synthesis pathway (Lorenz and Fink, 2001). Lorenz and Fink (2001) identified that the Icl1 mutant strain was unable to utilize acetate or ethanol. Icl was closely associated with the virulence of *C. albicans*, and the mutant which lacks the ICL1 gene was obviously less virulent compared with the wild type in mouse models (Lorenz and Fink, 2001; Barelle et al., 2006). Thus, it is worthy to do more researches on Icl, as shown in Figure 1, and currently the development of antifungal compounds based on this target has been carried out (Cheah et al., 2014; Ansari et al., 2018). Several studies have identified that apigenin, itaconic acid, monoterpenoid perillyl alcohol, and caffeic acid were potential Icl1 inhibitors against *C. albicans* with the MIC 125–1000 μg/ml, and although their antifungal activities were limited, they proved that the enzyme has potential therapeutic value for exploring new antifungal drugs (Cheah et al., 2014; Bae et al., 2015; Ansari et al., 2018). Bae et al. demonstrated that Mohangamide A and Mohangamide B also displayed essential antifungal effects; they could inhibit *C. albicans* Icl activity with the IC_50_ 4.4 and 20.5 μM, respectively (Bae et al., 2015). These accumulating evidences suggest that doing researches on Icl is valuable, and we believe that continuing to develop more details about this target will reveal new and effective antifungal drugs.

### Malate Synthase

Malate synthase (Mls) encoded by MLS1 gene is another unique enzyme in the glyoxylate cycle, which converts acetyl-CoA and glyoxylate to malate in the glyoxylate cycle (Lorenz and Fink, 2001). Like the other specific enzyme Icl in the glyoxylate cycle, MLS not only participates in the glyoxylate cycle but also is indispensable for *C. albicans* virulence (Lorenz and Fink, 2001; Ansari et al., 2018). The virulence and persistence in systemic candidiasis murine models infected with MLS1 gene mutant was decreased compared with wild-type strains, demonstrating that MLS1 does have influences on *C. albicans* virulence (Lorenz and Fink, 2001; Ansari et al., 2018). In *Paracoccidioides*, Mls also played an important role in adhesion and host–fungus interactions, and four alkaloid compounds could inhibit Mls of *Paracoccidioides* with IC_50_ 17–29 μg/ml, which had obvious effects on fungal infections (Costa et al., 2015). *C. albicans* Mls also shares significant homology with a lot of microorganisms as well as plants, but not humans, indicating that antifungal compounds based on Mls could have broad antimicrobial effects and limited side effects on human bodies (Lorenz and Fink, 2001). Therefore, although the antifungal compounds based on *C. albicans* Mls have not been studied yet, we believe that the accumulated evidence suggests that this enzyme is valuable and meaningful for the development of novel antifungal drugs.

### UDP-Galactose-4-Epimerase

UDP-galactose-4-epimerase (GAL10) is a key enzyme in the galactose metabolism which is responsible for converting UDP-galactose to UDP-glucose and then can be used for subsequent glycolysis (Singh et al., 2007). Comparing with other enzymes in *C. albicans* glucose metabolism, GAL10 not only is involved with hyphae formation but also has influences on the colony morphology (Singh et al., 2007). Singh et al. confirmed that the colony morphology had more wrinkles and the hyphal formation was increased in null GAL10 mutant compared with the wild-type strains (Singh et al., 2007). Moreover, GAL10 also plays a positive role in improving the sensitivity of antifungal drugs. GAL10 mutant showed hypersensitivity to antifungal agents and oxidative stress conditions (Singh et al., 2007). Thus, these accumulated evidences may prove that GAL10 is possible to be a potential antifungal target theoretically; it needs further exploration to determine the possibility.

### HXK2

Glycolysis is important for *C. albicans* to develop infections in plasma and tissues due to the ability of assimilating six-carbon compounds; HXK2 is one of the hexokinases in *C. albicans*, and it is responsible for the important step of glycolysis pathway, hexose phosphorylation (Eschrich et al., 2002; Barelle et al., 2006; Askew et al., 2009; Strijbis and Distel, 2010; Laurian et al., 2019). HXK2 contributes to *C. albicans* hexose phosphorylation and invasion abilities including filamentation and virulence (Laurian et al., 2019). The hxk2Δ/Δ mutant not only exhibited an obvious hyphal formation defect compared with the wild-type strains but also displayed much lower virulence in *Galleria mellonella* models and macrophage models, indicating that HXK2 indeed plays an essential role in *C. albicans* infections (Laurian et al., 2019). Moreover, HXK2 is closely related to physiological process such as high glycolytic metabolism, cell proliferation, apoptosis, and autophagy in tumor cells, and the expression of HXK2 is significantly increased in cancer cells compared with normal cells, so the inhibition of HXK2 is an effective antitumor strategy (Vander Heiden et al., 2009; Wolf et al., 2011; Zhang et al., 2014). In terms of the structure of hexokinases, the mammalian enzyme lacks a segment which corresponds to approximately 35 residues at the *Saccharomyces cerevisiae* enzyme (Wilson, 1995). Although research on antifungal drugs based on this target has not progressed yet, these accumulated evidences and structure differences may prove that HXK2 has the possibility to be a potential antifungal target theoretically, but it needs further exploration to determine the feasibility.

### Glyceraldehyde-3-Phosphate Dehydrogenase

Glyceraldehyde-3-phosphate dehydrogenase (GAPDH) is an essential glycolytic enzyme located in cytosol which consisted of four subunits; it is responsible for converting glyceraldehyde-3-phosphate to glycerate-1,3-bisphosphate. Except for its significant role in the process of glycolysis in *C. albicans*, GAPDH has been identified as the cytosolic antigen on the cell wall surface which also is enzymatically active (Gil-Navarro et al., 1997). Gil et al. (1999) and Delgado et al. (2003) suggested that the cell wall-bound GAPDH expressed both on the surface of clinical isolates and on the fungal cells in infected host tissues. Generally, adhesion is associated with tissue invasive process and virulence in *C. albicans* (Gil et al., 1999). Because of the ability that GAPDH in *C. albicans* can bind hosts’ fibronectin and laminin, it contributes to the progress of adhesion during candidiasis infections (Gil et al., 1999). Moreover, GAPDH was reported to be found in the outermost layers of the cell wall, and the anti-GAPDH IgG antibody plays an important role in defining candidiasis infections (Seidler, 2013). In theory, GAPDH is possible to be regarded as a potential antifungal target, and it needs further experimental researches to identify the feasibility.

### Enolase

Enolase, also called 2-phospho-D-glycerate hydrolyase, is one of the central enzymes of glycolysis pathway, catalyzing the dehydration of 2-phosphoglycerate to create phosphoenolpyruvate (Sundstrom and Aliaga, 1992; Sundstrom and Aliaga, 1994). Vertebrates contain three kinds of enolases, while *C. albicans* and *Candida glabrata* cells have only one enolase, Eno1 (Sundstrom and Aliaga, 1994). Eno1 is an immunodominant enzyme which is reported to exist in the inner layers of the cell wall abundantly in *C. albicans* since it shows humoral and cell-mediated immune response (Sundstrom and Aliaga, 1994; Angiolella et al., 1996). It has been confirmed that *C. albicans* eno1/eno1 null mutant could not grow on a glucose-containing medium, but it was capable of growing on non-fermentable carbon sources containing media, demonstrating that Eno1 is responsible for implicating in glucose metabolism (Ko et al., 2013). Besides, Jong et al. (2003) suggested that *C. albicans* Eno1 is associated with the progress of adhesion, and Eno1 could bind plasminogen and plasmin of host niches, which improved the process of invasion to human brain microvascular endothelial cells. Also, Eno1 not only has influence on host–fungus interactions but also contributes to the sensitivity of antifungal drugs, hyphae formation, and virulence (Ko et al., 2013). The eno1/eno1 mutant exhibited higher susceptibility to classical antifungal agents, a remarkable reduction in hyphal formation, and a noticeable decrease in pathogenicity in mice models compared with the wild-type strains (Ko et al., 2013). Furthermore, the homology similarity of Eno1 among opportunistic pathogenic fungus such as *C. albicans*, *Aspergillus fumigatus*, and *Cryptococcus neoformans* is relative high, so the inhibitors against this target will have a broad spectrum of antifungal activities (Ko et al., 2013). Several reports identified that *C. albicans* Eno1 is the major cell surface antigen and it existed on the outermost layers of the cell wall (Pitarch et al., 2014). Eno1-IgG antibodies exist in patients with candidemia, so monoclonal antibodies against Eno1 may become one of the promising strategies for *C. albicans* infection (Pitarch et al., 2014).

### Fructose-1,6-Bisphosphate Aldolase

Fructose-1,6-bisphosphate aldolase (Fba1p) is an essential enzyme to keep glucose balance both in glycolysis and gluconeogenesis which catalyzes the reversible cleavage reaction of fructose-1,6-bisphosphate (FBP) into two trioses, dihydroxyacetone phosphate (DHAP) and glyceraldehyde-3-phosphate (G3P) (Rodaki et al., 2006). Fba1p has two mechanically distinct forms: class I Fbas (FBA-I) and class II Fbas (FBA-II). FBA-I is generally found in higher organisms (mammals and plants) and some prokaryotes which forms a covalent Schiff-base intermediate between the dihydroxyacetone moiety of the keto substrate (FBP or DHAP) and a lysine residue of the active site (Marsh and Lebherz, 1992; Daher et al., 2010). FBA-II which is encoded by FBA1 gene exists predominantly in the pathogenic microbes in homodimeric form such as fungus and parasites, and they also require a divalent ion (generally Zn^2+^ or Fe^2+^) to maintain the stability of the intermediate metabolites during the catalytic activity and polarize the keto carbonyl group of substrate of the reaction (Daher et al., 2010; Han et al., 2017; de Amorim et al., 2018). In addition, *C. albicans* FBA1 gene and its orthologs in several fungus including *Schizosaccharomyces pombe*, *Neurospora crassa*, and *Aspergillus nidulans* show a strong genome sequence conservation, so the inhibitors directly against *C. albicans* FBA-II may have broad antifungal activities against some other fungus (Rodaki et al., 2006). Like most other enzymes in *C. albicans* glucose metabolism, FBA-II not only participates in glucose metabolism but also connects closely with adhesion and virulence (Crowe et al., 2003; Labbe et al., 2012). FBA-II was one of the binding proteins of *C. albicans*; the virulence would be impeded if FBA-II was inhibited, elucidating that FBA-II indeed plays a vital role in *C. albicans* virulence (Crowe et al., 2003; Labbe et al., 2012). The relationship between *C. albicans* growth and FBA-II has also been confirmed. Rodaki et al. suggested that FBA-II is closely related to the growth of *C. albicans*, and they identified that if the growth is inhibited remarkably, the FBA-II must be dropped to a very low level (Rodaki et al., 2006). However, the loss of FBA-II prevented the growth of *C. albicans* but did not kill it, and the virulence of FBA-II deletion strain partially existed but not eliminated absolutely according to the mouse model of systemic infection (Rodaki et al., 2006; Labbe et al., 2012). So FBA-II is needed to do further researches to explore more aspects, and in Figure 1, there are antifungal compounds against this target according to some researches.

Several reports identified that *N*-(4-hydroxybutyl)-glycolohydroxamic acid bis-phosphate is the FBP analog which has the 10^5^ selectivity to inhibit the FBA-II, and the IC_50_ value is 0.003 μg/ml (Daher et al., 2010). Also, other synthetic compounds of FBP analogs are *N*-(4-hydroxybutyl)-phospho-glycolohydroxamic acid, its hexanoylester, and its lauroylester, which also show strong inhibitory activity and selectivity toward FBA-II, with IC_50_ 0.3, 0.28, and 0.8 μg/ml, respectively (Daher et al., 2010). In addition, it has been found that some designed derivatives upon phenylhydrazone against FBA-II especially in *C. albicans* have been identified, such as the compound 3g (mentioned in this review is compound A); compound A is the most potent inhibitor with an IC_50_ value of 2.7 μg/ml, and the IC_50_ value of compound A was decreased from 2.7 to 0.063 μg/ml when combined with 8 μg/ml fluconazole, which showed an obvious synergistic inhibitory activity against resistant *C. albicans* (Han et al., 2017). The accumulating evidence indicated the importance of exploiting the FBA-II inhibitors for antifungal treatment, and we believe that there will be more new effective antifungal drugs produced based on the structure of this target.

### Pyruvate Kinase

Pyruvate kinase (Pyk) is an important enzyme for converting phosphoenolpyruvate (PEP) to pyruvate and ATP in *C. albicans* glycolysis metabolism. The existing form of Pyk is non-identical among mammals, prokaryotes, and lower eukaryotes; there are four distinguishing isoenzymes in mammals, while most prokaryotes and lower eukaryotes have only one isoenzyme, so the inhibitors against Pyk will have limited side effects on humans (Labriere et al., 2017). Except for the importance of the glycolysis pathway, Pyk also has a greater impact on *C. albicans* virulence (Barelle et al., 2006). Barelle et al. (2006) suggested that the virulence of *C. albicans* pyk1/pyk1 null mutant was attenuated *in vivo*, and the degree of virulence reduction was more obvious than ICL1/ICL1 mutant or pck1/pck1 strain. In conclusion, the current research on this target is not thorough enough and antifungal compounds have not been discovered, but the evidence indicates that this target is worthy to doing further researches, and we believe that Pyk is a potential good target for developing new antifungal agents.

### Glucosamine-6-Phosphate Synthase

Glucosamine-6-phosphate (GlcN-6-P) synthase catalyzes the irreversible step in UDP–GlcNAc biosynthetic pathway, which transforms D-fructose-6-phosphate (Fru-6-P) to D-glucosamine-6-phosphate (Janiak et al., 2003; Milewski et al., 2006). GlcN-6-P synthase also exists in mammals, but the inhibition of the enzyme in fungi and mammals has different physiological consequences, so specific inhibitors against GlcN-6-P synthase have little side effects on humans (Janiak et al., 2003). Except for playing a vital role in chitin biosynthesis pathway, GlcN-6-P synthase also had close relationships with hyphae formation (Gabriel et al., 2004). Several reports identified that the loss of GlcN-6-P synthase phosphorylation by protein kinase A obviously decreases but not completely eliminates the *C. albicans* germination (Gabriel et al., 2004), so the phosphorylation of GlcN-6-P synthase is important for germination and mycelial growth of *C. albicans*.

As shown in Figure 1, currently the development of antifungal compounds based on GlcN-6-P synthase has been carried out. Derivatives of *N*^3^-(4-methoxyfumaroyl)-(*S*)-2,3-diaminopropanoic acid (FMDP) which have great antifungal activities targeted GlcN-6-P synthase have been synthesized, including compounds 4, 5, 11, 12, and 13 (mentioned in this review is FMDP derivatives 1, 2, 3, 4, and 5), with MIC of 4, 32, 0.25, 0.5, and 0.5 μg/ml, respectively (Pawlak et al., 2015, 2016). These findings indicated the significance of exploiting GlcN-6-P synthase inhibitors for antifungal treatment, and we believe that there will be more new effective antifungal drugs produced based on this target.

### GlcN-6-P Acetyltransferase

GlcN-6-P acetyltransferase is the second enzyme encoded by GNA1 gene in UDP–GlcNAc biosynthetic pathway; it is responsible for converting acetyl-CoA and GlcN-6P into CoA and GlcNAc-6P (*N*-acetylglucosamine-6-phosphate) (Hurtado-Guerrero et al., 2008). Several reports pointed that GNA1 gene was efficient for *C. albicans* growth and virulence. The gna1Δ mutant could grow normally in media containing GlcNAc, but it dramatically enlarged, expanded and could not be separated in media without GlcNAc (Mio et al., 2000; Milewski et al., 2006). Furthermore, the gna1Δ mutant exhibited a reduced virulence in the mouse model of candidiasis compared with wild-type strains, indicating that GNA1 gene is important to *C. albicans* virulence (Mio et al., 2000; Milewski et al., 2006). Also, several studies identified the structural difference of the enzyme between fungi and mammals. There is a fungal selective pocket which only positions in fungi, so specific inhibitors against GlcN-6-P acetyltransferase may have little side effects on humans (Lockhart et al., 2020). Therefore, GlcN-6-P acetyltransferase may have the possibility to be a potential antifungal target theoretically, but it still requires a lot of in-depth researches to determine the feasibility.

## Discussion

As shown in Table 1, *C. albicans* adhesion, hyphal formation, and virulence are closely linked to several enzymes in glucose metabolism pathways, and it has been proved that some compounds exert antifungal activities and/or increase the sensitivity to traditional antifungals by inhibiting these enzymes. In addition, several enzymes among these potential antifungal targets are absent in humans or the inhibition of enzymes in fungi and mammals has different physiological consequences, indicating that the specific inhibitors acting on these targets will have little side effects on humans. We believe that by understanding more details between glucose metabolism and *C. albicans*, it will make a lot of sense for reversing drug resistance and developing new drugs against fungal infections.

## Author Contributions

XC wrote the review and created the Figure 1 and Table 1. SS, ZZ, ZC, and YL helped with it. SJS contributed to the writing of this article.

## Conflict of Interest

The authors declare that the research was conducted in the absence of any commercial or financial relationships that could be construed as a potential conflict of interest.
